# Metals in Particulate Pollutants Affect Peak Expiratory Flow of Schoolchildren

**DOI:** 10.1289/ehp.9531

**Published:** 2006-12-11

**Authors:** Yun-Chul Hong, Seung-Sik Hwang, Jin Hee Kim, Kyoung-Ho Lee, Hyun-Jung Lee, Kwan-Hee Lee, Seung-Do Yu, Dae-Seon Kim

**Affiliations:** 1 Department of Preventive Medicine, Seoul National University College of Medicine, Seoul, Republic of Korea; 2 Institute of Environmental Health, Seoul National University Medical Research Center, Seoul, Republic of Korea; 3 Department of Epidemiology and Biostatistics, Seoul National University School of Public Health, Seoul, Republic of Korea; 4 Department of Occupational and Environmental Medicine, Inha University Hospital, Incheon, Republic of Korea; 5 Division of Environmental Epidemiology, National Institute of Environmental Research, Incheon, Republic of Korea

**Keywords:** air pollution, genetic polymorphism, lung function, metals, particles

## Abstract

**Background:**

The contribution of the metal components of particulate pollutants to acute respiratory effects has not been adequately evaluated. Moreover, little is known about the effects of genetic polymorphisms of xenobiotic metabolism on pulmonary function.

**Objectives:**

This study was conducted to assess lung function decrement associated with metal components in particulate pollutants and genetic polymorphisms of glutathione *S-*transferase M1 and T1.

**Methods:**

We studied 43 schoolchildren who were in the 3rd to 6th grades. Each student measured peak expiratory flow rate three times a day for 42 days. Particulate air concentrations were monitored every day, and the concentrations of iron, manganese, lead, zinc, and aluminum in the particles were measured. Glutathione *S*-transferase M1 and T1 genetic polymorphisms were determined using DNA extracted from participant buccal washings. We used a mixed linear regression model to estimate the association between peak expiratory flow rate and particulate air pollutants.

**Results:**

We found significant reduction in the peak expiratory flow rate after the children’s exposure to particulate pollutants. The effect was shown most significantly 1 day after exposure to the ambient particles. Manganese and lead in the particles also reduced the peak expiratory flow rate. Genetic polymorphisms of glutathione *S*-transferase M1 and T1 did not significantly affect peak expiratory flow rate.

**Conclusions:**

This study demonstrated that particulate pollutants and metals such as manganese and lead in the particles are associated with a decrement of peak expiratory flow rate. These effects were robust even with consideration of genetic polymorphisms of glutathione *S*-transferase.

Many epidemiologic studies have reported an acute impact of particulate air pollutants on the pulmonary system, including the reversible decrement of pulmonary function and the increase of bronchial hyperreactivity (Boezen et al. 1998; Sharma et al. 2004; Ward and Ayres 2004). However, the biologic mechanism associated with particulate air pollutants has not been explained clearly. Reactive oxygen species (ROS) have been proposed as a potential pathway for the adverse biologic effects of particles (Donaldson et al. 2003). Several components such as transition metals, organic constituents, endotoxins, and acid sulfates have been postulated as participating in the biologic response (Ghio and Cohen 2005). Therefore, acute respiratory effects caused by particulate air pollutants may be attributed partly to metal elements causing damage by the generation of free radicals (Prahalad et al. 2000, 2001; Roemer et al. 2000). Subsequent events may include epithelial damage, increased permeability, and an inflammatory response leading to the decrement of lung function (Bergamaschi et al. 2001). However, the contribution of particulates’ metal components to acute health effects has not been adequately evaluated to date (Roemer et al. 2000).

Two European studies have reported that airborne iron was possibly associated with a decline in peak expiratory flow rate (PEFR), production of phlegm, or exacerbation of respiratory symptoms (Dusseldorp et al. 1995; Roemer et al. 2000). Because Fe of airborne particles was known to cause oxidative damage, other metals such as lead, manganese, or aluminum contained in particulate air pollutants could also generate oxygen free radicals leading to pulmonary injury (Gutteridge et al. 1996). In contrast, zinc has been known for its role in preventing free radical formation, so it could be related to protection from pulmonary injury (Stefanidou et al. 2006).

Members of the glutathione *S-*transferase (*GST*) super gene family are critical for protecting cells from the effects of ROS because they utilize, as substrates, a wide variety of oxidative stress products (Lee et al. 2004). Therefore, genetic polymorphisms associated with *GSTM1* and *T1* may affect pulmonary function because of different ability to scavenge ROS. The *GSTM1* and *T1* genes are deleted in approximately half of Asian populations (Kawai et al. 2005; Xu et al. 2005; You et al. 2005).

To determine whether exposure to metals in particulate matter (PM) and genetic polymorphisms of *GSTM1* and *GSTT1* were associated with PEFR in schoolchildren, we performed a panel study that included daily measures of the PEFR and PM concentrations. PEFR monitoring has been used for assessment of particulate air pollutant effects on airways because it is easy to perform and allows for a large number of measurements during the study period (Bellia et al. 2003). In a panel study with daily measurements of PEFR, each subject can be used as his or her own control, and only time-varying covariates for the subject need to be considered in the analysis. We hypothesized that elevations of PM or metal components in PM are associated with a decrease of PEFR in schoolchildren, and that genetic polymorphisms of *GSTM1* and *GSTT1* affect PEFR as well.

## Materials and Methods

### Study population

The study group consisted of children in a school on the Dukjeok Island near Incheon City, Korea. We invited all of the 46 students in the 3rd to 6th grades to participate. Because three students declined participation, we studied 43 schoolchildren from 23 March to 3 May 2004. A face-to-face survey with a standardized questionnaire was performed on the first day. We collected information about medical history of asthma, rhinitis, or eczema; passive smoking exposure at home; family history of pulmonary disease; socioeconomic status; household environment, including heating and cooking sources; and exposure to pets. Because the school was located on an island where traffic density and industrial emissions are low, natural sources including dust from the deserts of Mongolia or China may also have contributed to the metal levels besides traffic or industrial sources. The study protocol was approved by the institutional review board at Inha University Hospital, and written informed consent was obtained from the parents of all study participants.

### Lung function measurement

On the first day, each student was asked to perform three maximum forced expiratory flow-volume tests using a flow spirometer (Medgraphics, St. Paul, MN, USA), from which the forced vital capacity (FVC) and forced expiratory volume in 1 sec (FEV_1_) were recorded. The best performance from three trials was used as the baseline for lung function in the analysis. Each student was provided with a peak expiratory flow meter (Clement Clarke International Ltd., Essex, UK) to measure the PEFR three times per day at 0900, 1200, and 2000 hr daily during the study period. For each measurement, students took the best of three readings from the PEFR for analysis. The PEFR data from the first 7 days were not used for data analysis because a training period was needed to learn the PEFR measuring technique. The morning and daily mean PEFR data were used for the final analysis because the morning PEFR represented the effect of air pollutants during the night and early morning, and the daily mean PEFR represented the effect during the day.

### Monitoring of ambient air pollutants and metal analysis

To estimate the daily exposure to particulate air pollutants, we measured ambient levels of PM with aerodynamic diameter < 2.5 μm (PM_2.5_) and < 10 μm (PM_10_) on the rooftop of an office building 2 km away from the school during the study period. The dust on the polytetrafluoroethylene filters was gravimetrically analyzed to obtain average daily concentrations of PM_2.5_ and PM_10_. Metal components from the collected PM_10_ were analyzed to evaluate the association between metal concentrations and lung function decrement. The concentrations of Fe, Mn, Pb, Zn, and Al from the collected PM_10_ were determined by use of an inductively coupled plasma-mass spectrometer (HP 4500; Hewlett Packard, Wilmington, DE, USA). We calculated the concentrations used in the analysis as the ratio of the metal amount in the PM_10_ sample to the air volume collected during the sampling. Data on 24-hr average temperature, relative humidity, and air pressure were obtained from the Korea Meteorological Administration (www.kma.go.kr).

### Genotyping

The participants were asked to collect mouthwash samples at the baseline health examination. They rinsed their mouth twice with 50 mL phosphate buffered saline (PBS), swished 30 mL mouthwash containing PBS throughout the mouth for 60 sec, and delivered the expectorant into a 50-mL tube. Cells were collected by centrifugation and then genomic DNA was isolated from a cell pellet using QIAamp DNA Mini Kit (Qiagen GmbH, Hilden, Germany). We analyzed the cells for genetic polymorphisms by polymerase chain reaction in a PTC-200 thermal cycler (MJ Research, Watertown, MA, USA), as described previously (Hong et al. 2000). The repeatability test was conducted for five samples for each genotyping (> 10% of all samples), resulting in a 100% concordance rate.

### Statistical analysis

We assessed associations of PM exposure, individual genetic status for *GSTM1* and *GSTT1* polymorphisms, and daily mean or morning PEFR. Medical history of asthma, rhinitis, or eczema; passive smoking exposure at home; family history of pulmonary disease; socioeconomic status; household environment including heating and cooking sources; and exposure to pets did not significantly affect PEFR measurements. We made a final statistical model with asthma history and passive smoking exposure at home among these factors with individual characteristics. We estimated least-square means of PEFR after controlling for age, sex, height, weight, asthma history, and passive smoking exposure at home. We used linear mixed-effects models to estimate the particulate pollutant effects on the daily PEFR, controlling for individual and meteorologic variables. Because the distributions of metal concentrations in PM_10_ were skewed, we used log-transformed data for these measurements in the linear mixed models. Genotype was also included in the model for evaluation of the effect of *GSTM1* and *GSTT1* polymorphisms. We treated age, sex, height, weight, asthma history, passive smoking exposure at home, genotypes, particulate pollutant concentrations, temperature, relative humidity, air pressure, and day of the week as fixed effects. Each student was treated as a random effect in the models.

## Results

We studied 43 participants enrolled in the 3rd to 6th grades at an elementary school. As shown in Table 1, there were 23 boys and 20 girls among the participants. The average value for PEFR, FVC, and FEV_1_, genotypes of *GSTM1* and *GSTT1*, as well as other summary statistics for the group are given in Table 1. Because genotyping from one student was not available, *GSTM1* and *GSTT1* genotypes for 42 students are shown. Null genotype frequencies for *GSTM1* were 22.7% for male and 70.0% for female students (*p* < 0.01). Those for *GSTT1* were 45.5% and 65.0%, respectively (*p* = 0.20). Table 2 summarizes the levels of PM_2.5_ and PM_10_ with the metal concentrations, temperature, relative humidity, and air pressure. Analysis of Fe, Mn, Pb, Zn, and Al showed that Fe concentrations were the highest among the metals, followed by Al, Pb, Zn, and Mn.

In the analysis of the *GSTM1* or *GSTT1* polymorphism, the difference in the PEFR between the two genotypes did not reach statistical significance after adjusting for age, sex, height, weight, asthma history, and passive smoking exposure (Table 3).

**Figure 1:**
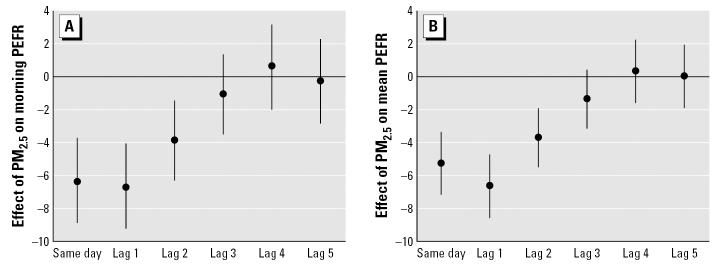
Lag distribution of morning PEFR (*A*) and daily mean PEFR (*B*) change by interquartile increase of PM_2.5._ Estimated decrements of PEFR (dots) and 95% confidence intervals (error bars) controlling for age, sex, height, weight, asthma history, environmental tobacco smoke exposure, meteorologic variables, and day of the week are shown.

Figure 1 shows the lag distribution of the PEFR change by interquartile range increase of PM_2.5_ from the current day to the 5 previous days. Considering the distribution of the lag effects of PM_2.5_ exposure, we chose a 1-day-lag model for further analysis.

One day after exposure to PM_2.5_, we found a significant decrease of the PEFR adjusting for age, sex, height, weight, asthma history, passive smoking exposure, meteorologic variables, and day of the week. The mean estimate of the decrement, for 1 μg/m^3^ PM_2.5_ 1 day before, was −0.54 L/min for the morning and the daily mean PEFR. Even though the effect of PM_10_ was not significant, the analysis of the metal composition of the PM_10_ showed that the regression coefficients for Mn and Pb, on the morning and the daily mean PEFR, were significantly negative. However, Fe, Zn, and Al concentrations were not significantly associated with the PEFR (Table 4).

When we analyzed the effect of the PM_2.5_, Pb, or Mn and genetic polymorphisms together in the multivariate model, PM_2.5_, Pb, and Mn affected the PEFR significantly and the regression coefficients changed little compared with those in the statistical model without genetic polymorphisms. The effects of the *GSTM1* or *GSTT1* polymorphism remained nonsignificant in the multivariate analysis (Table 5).

## Discussion

The present study demonstrates that metals in particulate pollutants as well as PM_2.5_ are associated with a decrement in the peak expiratory flow rate. Our hypothesis for lung function decrement associated with particulate air pollutants is that certain metal components in the particles may contribute to damage to the respiratory system via the generation of free radicals (Seaton et al. 1995). This study showed that particulate air pollutants, or some metals contained in the particles, significantly affected the PEFR after adjusting for confounding factors as well as the genetic polymorphisms of *GSTM1* and *GSTT1*.

Iron and other transition metals have been mentioned as elements that may be responsible for the effects of PM_10_ on respiratory health (Seaton et al. 1995). Because the presence of pro-oxidant Fe in normal lung fluid is an important factor that makes the lung vulnerable to oxidative stress, additional deposit of Fe or other metals through air-borne particles could lead to increased oxidative damage (Gutteridge et al. 1996). However, we did not find a significant relationship between Fe content in PM_10_ and decrement of lung function. This finding is inconsistent with reports linking Fe to oxidative damage or lung function, but other reports also showed that the iron content of PM_10_ or total suspended particles was less consistently associated with PEFR or mortality than were the particulate concentrations (Dusseldorp et al. 1995; Gutteridge et al. 1996; Hoek et al. 1997; Roemer et al. 2000).

Mn, one of the most abundant elements in the earth’s crust, has been known to have a dual effect as a pro-oxidant and as an antioxidant. Mn can produce free radicals at cytotoxic levels causing oxidative stress; therefore it can also modulate Fe-induced oxidation (HaMai and Bondy 2004; Han et al. 2005). Our findings showed that a decrement of the PEFR was significantly related to the concentration of Mn. In agreement with our results, Boojar and Goodarzi (2002) also reported that long-term exposure to Mn caused a significant decrease in pulmonary function.

Pb is found mostly as a product of industrial or combustion sources. Oxidative stress has been reported to be one of the important mechanisms underlying the toxic effects of Pb (Daggett et al. 1998). Studies on the production of ROS have suggested that exposure to Pb alters the status of ROS or oxidative stress leading to inflammatory reactions (Saxena and Flora 2004). Therefore, Pb is one of the elements responsible for the effects of particulate pollutants. In the present study, we found a significant decrease in the PEFR associated with the Pb concentration in particulate pollutants. Corresponding results were demonstrated in a study by Bagci et al. (2004), where pulmonary function in battery and exhaust workers, who inhaled Pb, were significantly impaired compared with control groups.

Al has also been known to cause an increase in oxidative stress and has a potential to accelerate Fe-induced lipid peroxidation in brain tissue (Campbell et al. 2004; Xie and Yokel 1996). In a study using rat model of short-term exposure to concentrated ambient particles, oxidative stress measured as *in situ* chemiluminescence was significantly associated with Al in the heart but not in the lung (Gurgueira et al. 2002). In our observational study, we could not find significant associations between Al concentrations in PM_10_ and PEFR of schoolchildren.

We found that Zn concentrations were not significantly associated with PEFR. Zn is an essential element for cell proliferation and differentiation, and is known to be an important element in preventing free radical formation (Stefanidou et al. 2006). The role of Zn in the protection from free radical injury may be attributed to maintaining an adequate level of metallothioneins, which are avid scavengers of free radicals, and prevent the interaction of chemicals with Fe to form free radicals (Coppen et al. 1988; Stefanidou et al. 2006; Tapiero and Tew 2003). However, in a panel study of patients with chronic obstructive pulmonary disease, Zn from inhaled particulate was associated with FVC and FEV_1_ decrement (Lagorio et al. 2006). Therefore, the role of Zn in particulate pollutants, whether it is pro-oxidant or antioxidant, needs to be confirmed by larger and more focused studies.

Particulate air pollutants, regardless of their chemical composition, are known to induce oxidative stress (Becker et al. 2005; Ghio and Cohen 2005). Therefore they are likely to induce the reduction of glutathione, an intracellular scavenger of endogenously generated oxidants and toxic electrophiles. Because GST catalyzes the conjugation of electrophiles with glutathione, the detoxification enzymes GSTM1 and T1 have a pivotal role in catalyzing the conjugation of glutathione to electrophilic substrates. (Rushmore and Pickett 1993). Therefore, they may protect against oxidative stress and inflammation through detoxification of endogenous or exogenous oxidant chemicals (Hakim et al. 2004; Hayes and Strange 1995). Although host antioxidant defenses such as GSTs detoxify ROS, individuals differ in their ability to deal with an oxidant burden, and such differences are, in part, genetically determined (Barnes 1990). The human GST isoenzymes *GSTM1* and *T1* are polymorphic. A deletion is responsible for the existence of a null form lacking enzyme function (Palli et al. 2005). Therefore, individual susceptibility to particulate air pollution may vary in association with the genetic polymorphisms (Lee et al. 2004; Schwartz et al. 2005). However, in the present study, the effect of the *GSTM1* or *GSTT1* polymorphism did not reach statistical significance, whereas PM_2.5_ or metals showed statistically significant effects on PEFR.

Several limitations of this study should be noted. Obviously the sample size of this panel study is small, but we recruited 93.5% from all eligible students in the school. We recruited panels of children including those either previously diagnosed with asthma or exposed to passive smoking at home. Even though there were reports of increased children’s asthma with passive smoking exposure and worsened impact of exposure to air pollutants on respiratory function among susceptible subjects such as asthmatics, we did not find statistically significant effects of passive smoking exposure or asthma history on PEFR (Gergen et al. 1998; Lagorio et al. 2006). When we evaluated interactive effects of particulate pollutants and these factors, we found no significant interactions between them either. The personal PM_2.5_ or PM_10_ exposures were not directly measured. Instead, we used monitoring data from samples collected at a rooftop of a building 2 km away from the school. This may have given inaccurate measures of exposures to particulate air pollutants. However, this kind of measurement error is likely to cause a bias toward the null hypothesis and underestimate the air pollutant effects (Zeger et al. 2000). We did not measure exposures to NO_2_ or ozone, which also may be responsible for decrement in lung function. Therefore, such gaseous pollutants could have affected the relationship between particulate air pollutants or metals and pulmonary function as confounders.

We found associations between PM_2.5_ or the metals such as Pb and Mn in PM_10_ and the PEFR in primary school–age children. We also found that PM_2.5_ affected pulmonary function more than PM_10_ in terms of decrement of the PEFR. However, we could not find a significant decline of the PEFR with increased PM_10_, whereas PM_2.5_ significantly decreased the PEFR. Our results for PM_2.5_ showed a greater decrease of the PEFR, −0.54 L/min for 1 μg/m^3^, than reports from other panel studies in children where mean estimates ranged from −0.05 to −0.28 L/min for 1 μg/m^3^ of PM_2.5_ (Ward and Ayres 2004). The difference might be caused by different composition of particulate pollutants.

We also examined the relationships between genetic polymorphisms of *GSTM1* and *T1* and the PEFR, but did not find a significant relationship between the polymorphisms and the PEFR. In addition, the effect of particulate pollutants on PEFR was not influenced by genetic polymorphisms in the statistical model considering both particulate pollutants and genetic polymorphisms.

In summary, our results showed that particulate air pollution was probably associated with reduction of PEFR in schoolchildren. We also found that metals such as Mn and Pb in the particulate pollutants were responsible for the effects on the PEFR. The effect of particulate air pollutants on the PEFR was robust even with consideration of genetic polymorphisms of *GSTM1* and *T1*.

## Figures and Tables

**Table 1:** Characteristics of study subjects and their lung function and genotypes [mean ± SD or no. (%)].

	Total (*n* = 43)	Male (*n* = 23)	Female (*n* = 20)	*p*-Value
Age (years)	9.6 ± 1.1	9.7 ± 1.2	9.5 ± 1.1	0.48
Weight (kg)	35.8 ± 9.7	36.4 ± 8.8	35.2 ± 10.8	0.70
Height (cm)	139.0 ± 9.4	139.8 ± 8.6	138.1 ± 10.5	0.57
Asthma	6 (14.0)	3 (13.0)	3 (15.0)	0.85
ETS exposure	15 (34.9)	10 (43.5)	5 (25.0)	0.20
Morning PEFR	325.4 ± 57.7	337.4 ± 57.8	310.2 ± 55.5	0.13
Mean PEFR	328.0 ± 58.7	340.1 ± 58.9	312.4 ± 56.2	0.13
FVC	2.1 ± 0.5	2.2 ± 0.5	1.9 ± 0.4	0.05
FEV_1_	1.8 ± 0.4	1.9 ± 0.5	1.7 ± 0.4	0.22
*GSTM1* null	19 (45.2)	5 (22.7)	14 (70.0)	< 0.01
*GSTT1* null	23 (54.8)	10 (45.5)	13 (65.0)	0.20

ETS, environmental tobacco smoke.

**Table 2:** Summary of PM_2.5_, PM_10_, and metal levels and meteorologic data.

Variable	No. of days	Mean ± SD	Minimum	Median	Maximum
PM_2.5_ (μg/m^3^)	35	20.27 ± 8.23	5.94	22.07	36.28
PM_10_ (μg/m^3^)	32	35.30 ± 23.48	12.24	29.36	124.87
Fe (μg/m^3^)	27	0.208 ± 0.203	0.061	0.112	0.806
Mn (μg/m^3^)	27	0.008 ± 0.005	0.000	0.007	0.019
Pb (μg/m^3^)	27	0.051 ± 0.031	0.011	0.051	0.155
Zn (μg/m^3^)	27	0.021 ± 0.021	0.006	0.013	0.112
Al (μg/m^3^)	27	0.085 ± 0.100	0.017	0.031	0.344
Temperature (°C)	35	10.0 ± 3.1	3.8	10.7	17.2
Relative humidity (%)	35	62.9 ± 12.7	31.8	61.5	88.6
Atmospheric pressure (hPa)	35	1014.0 ± 4.6	1002.6	1014.7	1021.6

**Table 3:** Morning and daily mean PEFR according to *GSTM1 and GSTT1* genotype.

	Morning PEFR			Mean PEFR		
Sample	LS means^a^	SE	*p*-Value	LS means	SE	*p*-Value
All (*n* = 43)						
*GSTM1*						
Null	303.7	15.9	0.24	306.1	15.7	0.27
Present	323.9	14.2		325.1	14.1	
*GSTT1*						
Null	314.4	14.5	0.87	316.3	14.4	0.90
Present	316.9	15.1		318.3	14.9	
Male (*n* = 23)						
*GSTM1*						
Null	295.9	24.6	0.14	298.5	23.3	0.16
Present	329.6	16.7		329.0	15.8	
*GSTT1*						
Null	315.9	22.9	0.67	316.8	21.5	0.66
Present	326.0	18.0		325.5	16.9	
Female (*n* = 20)						
*GSTM1*						
Null	293.0	24.2	0.49	295.8	24.44	0.50
Present	315.5	31.5		318.5	31.8	
*GSTT1*						
Null	301.1	23.3	0.88	303.4	23.5	0.94
Present	296.2	33.3		301.0	33.6	

a Least-square (LS) means adjusted by age, sex, height, weight, asthma history, and environmental tobacco smoke exposure.

**Table 4:** Regression coefficients of morning and daily mean PEFR on PM_2.5_, PM_10_, and metal components of PM_10_ using linear mixed-effects regression.

	Morning PEFR				Mean PEFR			
	Crude		Adjusted a		Crude		Adjusted	
Variable	β	*p*-Value	β	*p*-Value	β	*p*-Value	β	*p*-Value
Lag1 (PM_2.5_)	−0.14	0.12	−0.54	< 0.01	−0.15	0.02	−0.54	< 0.01
Lag1 (PM_10_)	−0.00	0.99	−0.04	0.37	0.00	0.93	−0.05	0.12
Lag1 (logFe)	−1.26	0.31	−3.24	0.13	−1.20	0.20	−2.37	0.15
Lag1 (logMn)	−4.40	< 0.01	−9.82	< 0.01	−4.05	< 0.01	−8.44	< 0.01
Lag1 (logPb)	−6.79	< 0.01	−6.83	< 0.01	−6.23	< 0.01	−6.37	< 0.01
Lag1 (logZn)	−0.55	0.71	−0.98	0.59	1.33	0.24	1.53	0.28
Lag1 (logAl)	−0.58	0.57	−2.22	0.25	−0.59	0.45	−1.48	0.32

a Adjusted by age, sex, height, weight, asthma history, environmental tobacco smoke exposure, temperature, relative humidity, atmospheric pressure, and day of the week.

**Table 5:** Regression coefficients of morning and daily mean PEFR on PM_2.5_, metal components of PM_10_, and *GSTM1* and *GSTT1* genotype using a linear mixed-effects regression.

	Morning PEFR		Mean PEFR	
Variable	β^a^	*p*-Value	β	*p*-Value
Lag_1_ (PM_2.5_)	−0.57	< 0.01	−0.56	< 0.01
*GSTM1*	20.04	0.25	18.75	0.28
Lag_1_ (logPb)	−7.26	< 0.01	−6.43	< 0.01
*GSTM1*	21.19	0.23	20.09	0.25
Lag_1_ (logMn)	−10.31	< 0.01	−8.66	< 0.01
*GSTM1*	21.02	0.23	19.84	0.25
Lag_1_ (PM_2.5_)	−0.57	< 0.01	−0.56	< 0.01
*GSTT1*	2.31	0.89	1.75	0.91
Lag_1_ (logPb)	−7.26	< 0.01	−6.43	< 0.01
*GSTT1*	2.07	0.90	2.39	0.88
Lag_1_ (logMn)	−10.32	< 0.01	−8.67	< 0.01
*GSTT1*	2.02	0.90	2.33	0.88

a Adjusted for age, sex, height, weight, asthma history, environmental tobacco smoke exposure, temperature, relative humidity, atmospheric pressure, and day of the week in the model.
